# Retroperitoneal ganglioneuroma with ectopic inferior vena cava invasion: a case report

**DOI:** 10.3389/fsurg.2026.1730386

**Published:** 2026-04-08

**Authors:** Baisheng Xu, Huanglin Duan, Hongbing Gao, Fengui Leng, Feng Wang, Hui Che, Liangfei Guo, Jianmiao Hu, Xu Leng, Junwei Gu, Wenqiang Dong

**Affiliations:** Department of Urology, The First People's Hospital of Xiushui County, Jiujiang, Jiangxi, China

**Keywords:** ectopic inferior vena cava, ganglioneuroma, retroperitoneal tumor, robotic surgery, tumor

## Abstract

Ganglioneuroma (GN) is a rare benign tumor from neural crest tissue, composed of mature Schwann cells, ganglion cells, and nerve fibers. These tumors most often arise in the retroperitoneal region (52%) and posterior mediastinum (39%), while extra-adrenal lesions account for 51% of abdominal GN cases. This paper presents a 66-year-old female with paroxysmal atrial fibrillation found to have a left retroperitoneal mass on imaging. A 9.5-cm tumor was fully resected using robot-assisted laparoscopy. Pathology confirmed GN with ectopic inferior vena cava (IVC) invasion. This case highlights the need to consider retroperitoneal GN in the differential diagnosis of vascular structures. In addition, three-dimensional reconstruction is valuable for surgical planning, and robot-assisted surgery is preferred for complex cases.

## 1 Introduction

GN is an extremely rare and slow-growing benign neurogenic tumor, accounting for approximately 0.1% to 0.5% of nervous system tumors (1). GN originates from sympathetic ganglion cells of neural crest tissue and can occur anywhere in the sympathetic nervous system (2); however, it is most commonly found in the mediastinum and retroperitoneal space (1). Additionally, GN may arise in less common locations, such as the tongue, bladder, uterus, bone, and skin (3–5). GN predominantly affects children and adolescents, with up to 60% of patients being younger than 20 years at the time of diagnosis. Furthermore, the incidence of GN is higher in women than in men (2). Patients with GN often present with no obvious symptoms, and many are diagnosed incidentally during routine physical examinations or imaging studies. A minority of patients may require treatment for complications arising from large tumor size and compression of adjacent organs or tissues.

Currently, the preferred treatment for GN is complete surgical resection. However, when the tumor is large and invades surrounding tissues and organs, leading to adhesions, surgical separation can be particularly challenging. In this paper, we discuss the diagnosis and treatment strategy for retroperitoneal GN through a complex case IVC anatomical variation and vascular invasion.

## 2 Case report

A 66-year-old woman presented with chest tightness, shortness of breath, and upper abdominal discomfort. Upon being diagnosed with paroxysmal atrial fibrillation at our hospital, a CT examination unexpectedly revealed a left retroperitoneal mass. The patient reported no significant pain or discomfort in the left upper abdomen or waist during routine activities. Her medical history included hypertension, coronary heart disease, and hyperthyroidism. Neurological examination did not reveal any notable abnormalities. Physical examination revealed normal vital signs, with no palpable masses in the waist or abdomen. Laboratory tests showed (shown in Table 1) potassium levels at 3.20 mmol/L (normal range: 3.48–5.5 mmol/L), brain natriuretic peptide (BNP) at 222.12 pg/mL (normal range: 0–100 pg/mL), and troponin I (cTn I) at 3.208 ng/mL (normal range: 0–0.04 ng/mL). Additionally, total triiodothyronine (T3) was 3.31 nmol/L (normal range: 0.92–2.79 nmol/L), total thyroxine (T4) at 16.10 μg/dL (normal range: 4.5–10.9 μg/dL), free triiodothyronine (FT3) at 13.31 pmol/L (normal range: 2.77–6.31 pmol/L), and free thyroxine (FT4) at 2.96 ng/dL (normal range: 0.89–1.76 ng/dL), we summarized them in Table 1. Notably, during her hospital admission for infusion, the patient experienced sudden chest tightness and profuse sweating; timely ECG monitoring revealed a rapid ventricular rate and atrial fibrillation, which led us to consider paroxysmal atrial fibrillation and heart failure. Furosemide and cedilanid were administered to control the ventricular rate. Additionally, the patient received 10 mg of methimazole twice daily for preoperative preparation. Echocardiography findings included left atrial enlargement, left ventricular wall thickening, and aortic valve calcification with increased forward blood flow velocity. Bilateral common carotid artery intima-media thickness measured 1.2 mm (normal range: 0.5–1.0 mm), with detectable plaque formation, the largest measuring 17 × 2.4 mm.

**Table 1:** Biochemical test.

Test items	Result	Normal range
potassium	3.20 mmol/L	3.48–5.5 mmol/L
brain natriuretic peptide	222.12 pg/mL	0–100 pg/mL
troponin I	3.208 ng/mL	0–0.04 ng/mL
total triiodothyronine	3.31 nmol/L	0.92–2.79 nmol/L
total thyroxine	16.10 *μ*g/dL	4.5–10.9 μg/dL
free triiodothyronine	13.31 pmol/L	2.77–6.31 pmol/L
free thyroxine	2.96 ng/dL	0.89–1.76 ng/dL

**Figure 1:**
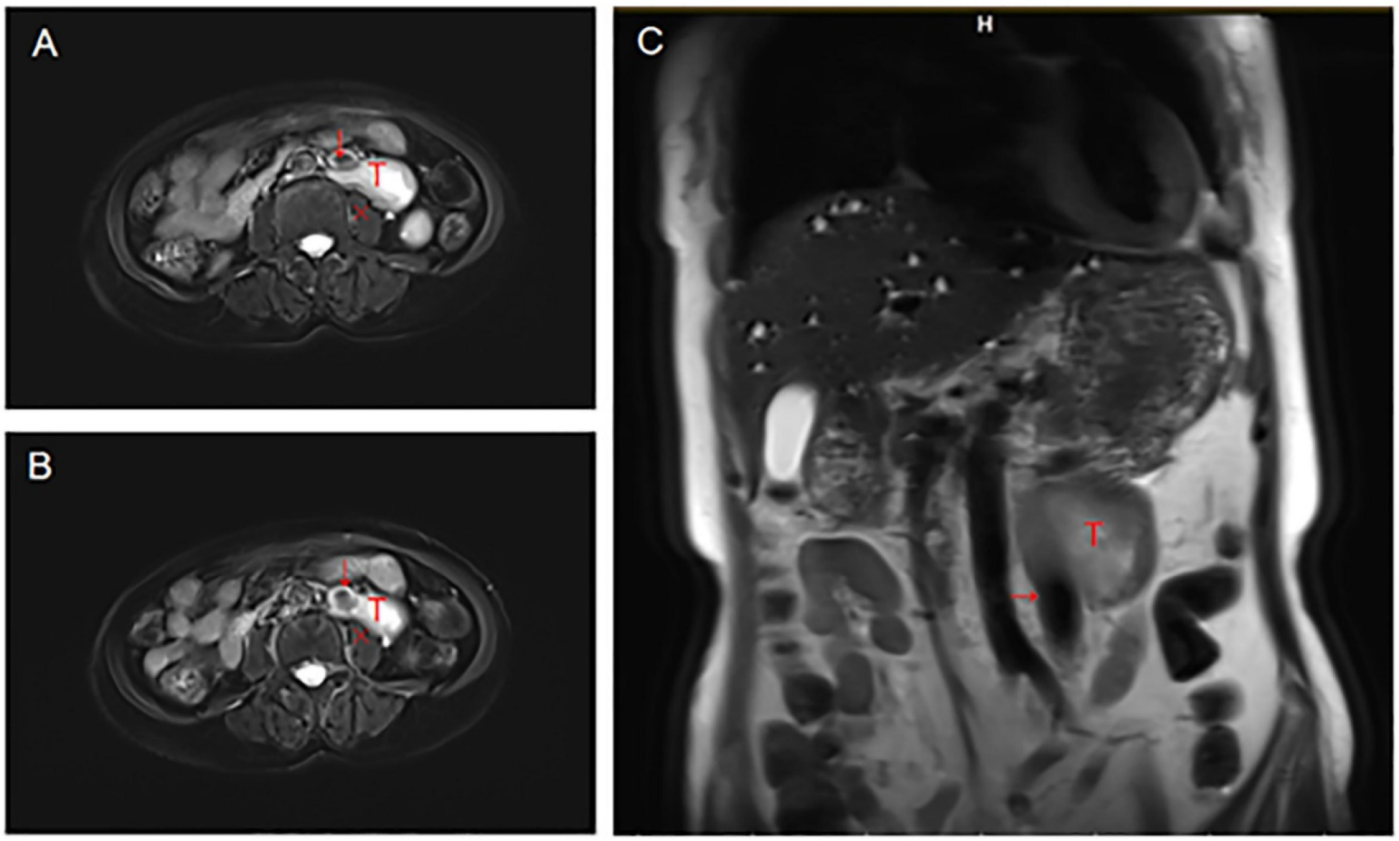
T2 MRI, **(A)** and **(B)** is transverse plane, **(C)** is coronal plane. Arrow indicates IVC, T indicates tumor, Fork indicates psoas muscle.

**Figure 2:**
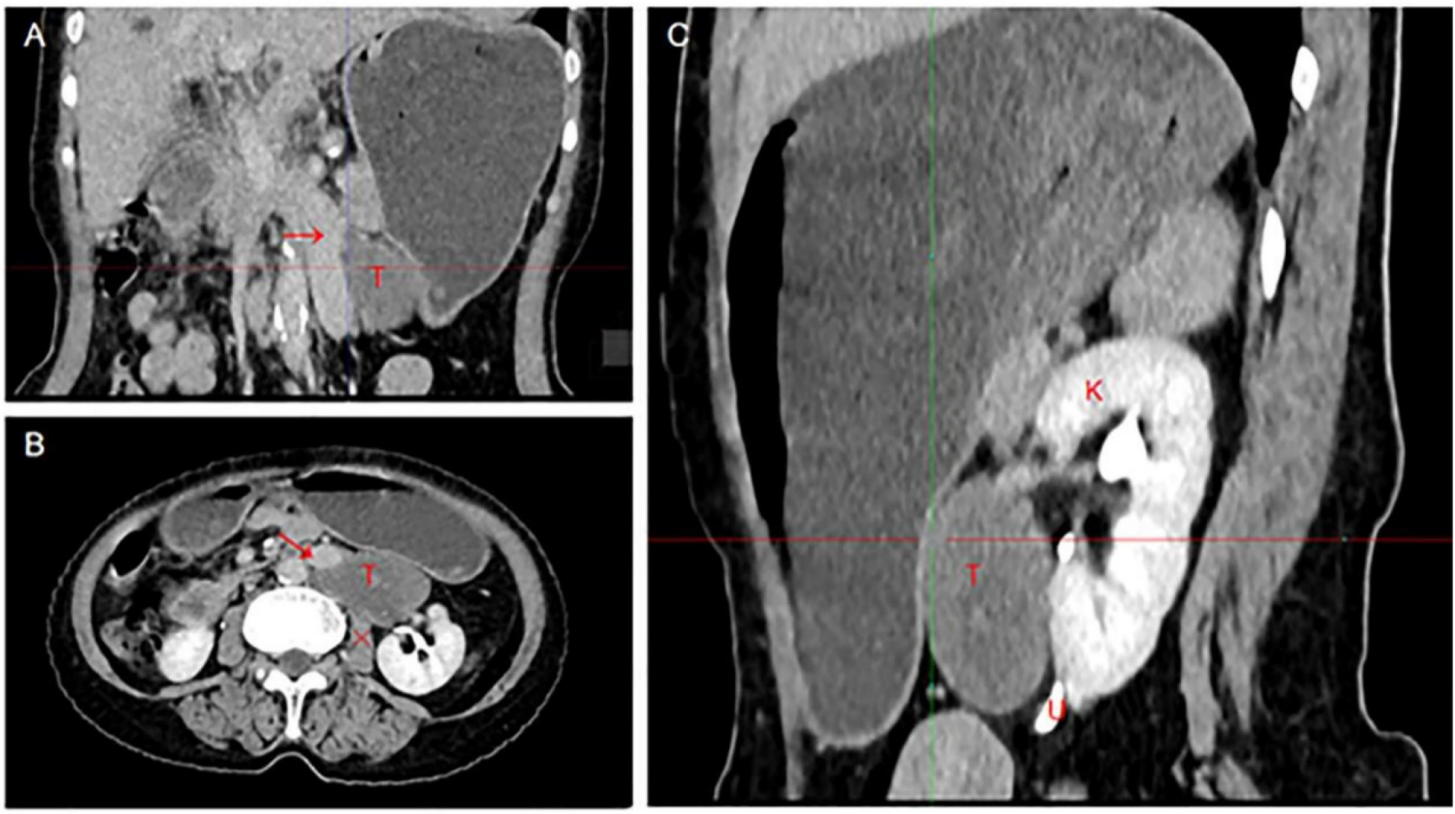
Enhanced CT image, **(A)** is coronal plane, **(B)** is transverse plane, **(C)** is sagittal plane. Arrow indicates IVC, T indicates tumor (CT attenuation values <40 HU), Fork indicates psoas muscle, K indicates kidney, and U indicates ureter.

In terms of imaging studies, enhanced CT and MRI examinations suggested a diagnosis of a retroperitoneal neurogenic tumor with smooth margins (shown in Figure 1 for MRI and Figure 2 for CT). However, considering the potential for the tumor to originate from the adrenal gland, we also evaluated levels of adrenocorticotropic hormone, cortisol, aldosterone, renin, and the aldosterone/renin ratio. The results indicated a slight elevation in supine aldosterone at 280.21 pg/mL (normal range: 29–240 pg/mL) and a mild increase in morning aldosterone between 7–10 AM at 132.15 pg/mL (normal range: 7–65 pg/mL), with no significant abnormalities observed in the other parameters.

**Figure 3:**
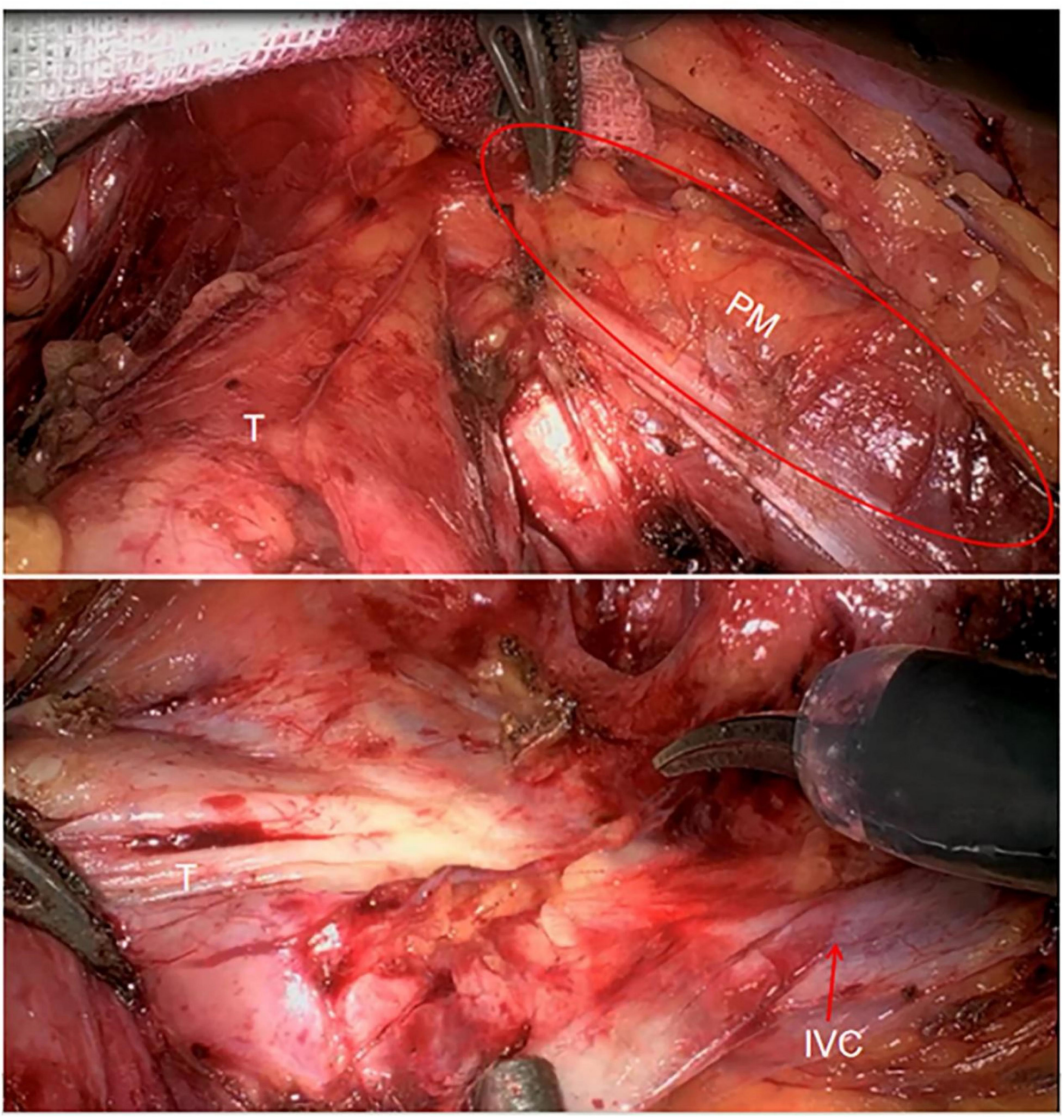
The relationship between tumor and psoas muscle and IVC during operation. T indicates tumor; PM indicates psoas muscle; IVC indicates inferior vena cava.

**Figure 4:**
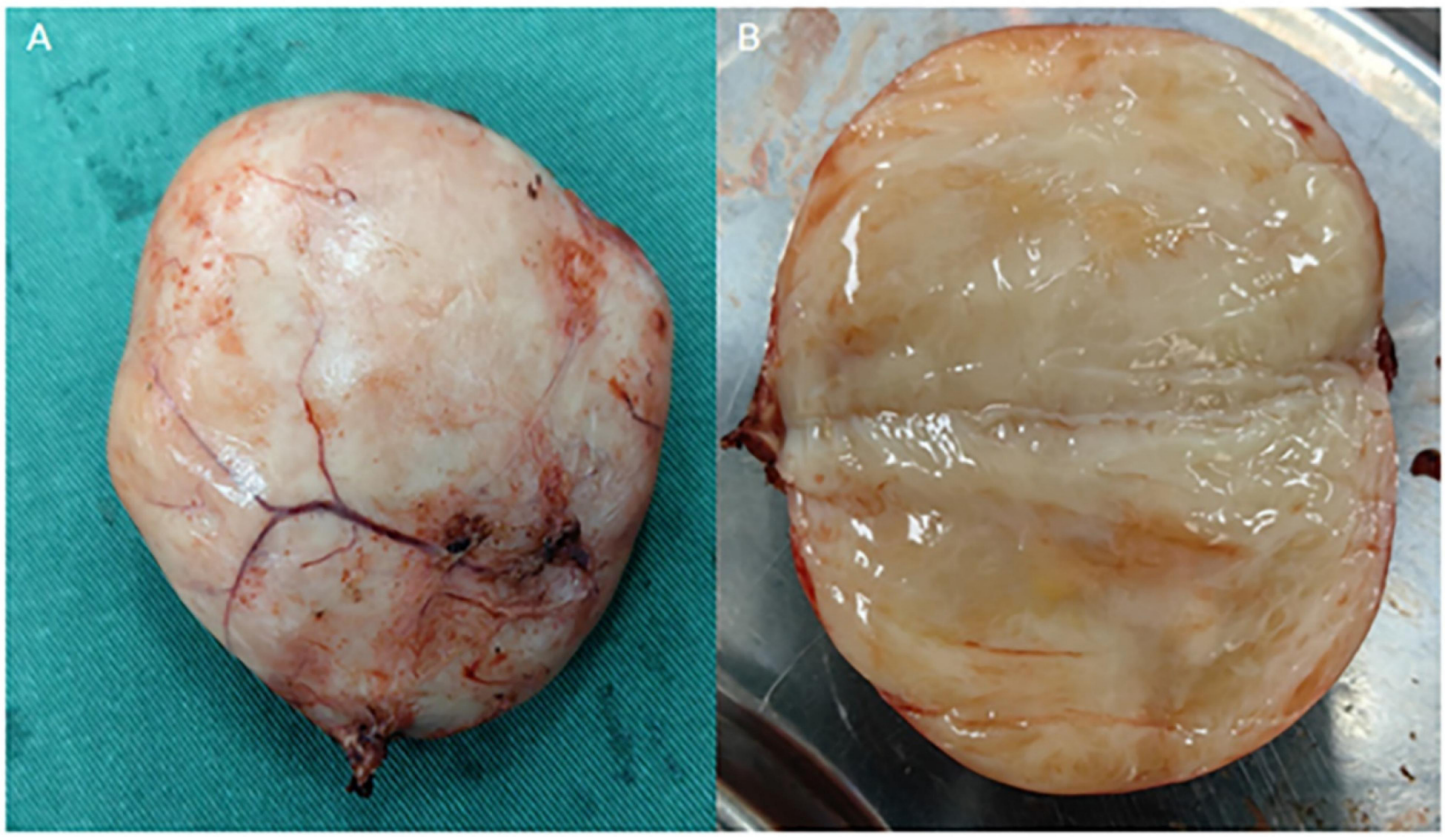
**(A)** specimen; **(B)** the specimen is locally mucus-like after cutting.

After stabilizing the patient's vital signs, we opted for robot-assisted laparoscopic approach for complete resection of the tumor. Initially, we separated the paracolic groove, exposing the surface of the tumor located on the left side of the retroperitoneum. We then dissected along the tumor's lower pole at the L2 vertebral level and noted its tight adhesion to the psoas muscle. Following the separation of the tumor from the psoas muscle (Figure 3 demonstrates), we flipped the tumor and observed that the ectopic inferior vena cava (IVC) was also adherent to it (Figure 3 demonstrates). IVC clamping was not required during vascular dissection. Ultimately, we managed to detach the tumor from both the psoas muscle and the ectopic IVC, allowing for its intact removal from the body. Upon gross examination, the tumor measured approximately 9.5 × 7 × 4 cm, with a cut surface displaying a grayish-white appearance and localized grayish-yellow foci (Figure 4 demonstrates).

**Figure 5:**
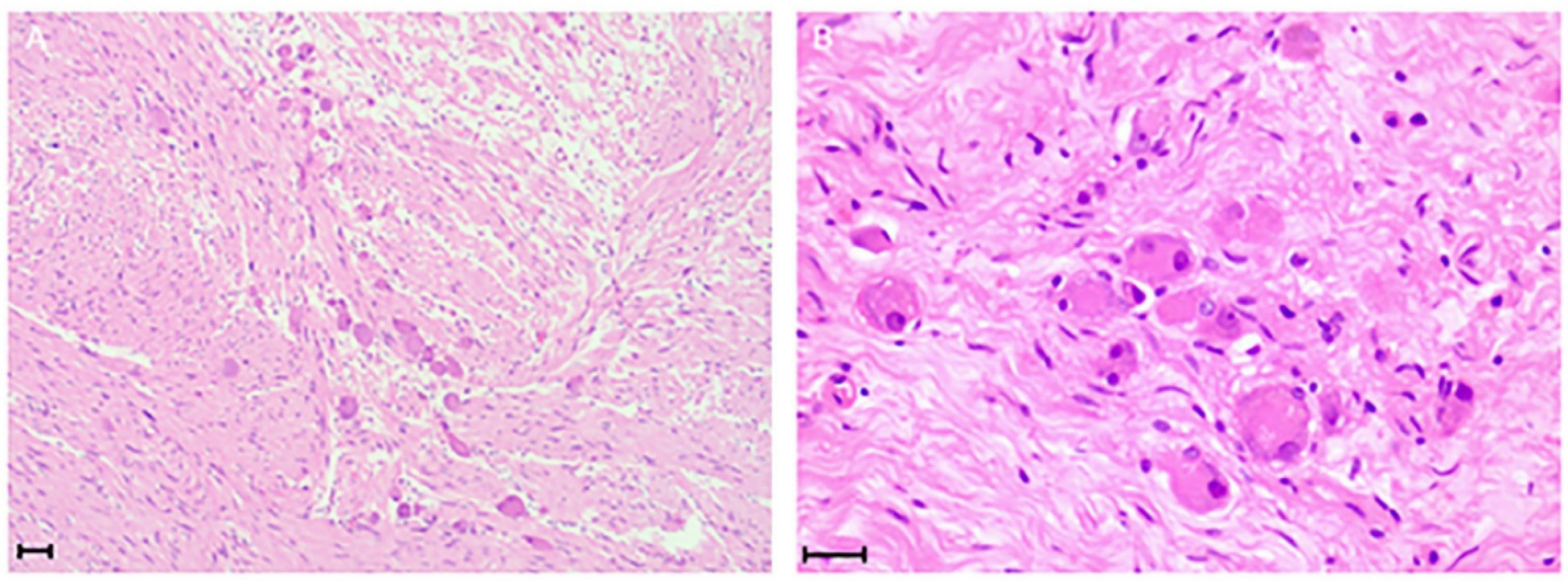
Spindle-shaped Schwann cells, round mature ganglion cells can be seen in 20× **(A)** and 40× **(B)** scale bar = 50 μm.

**Figure 6:**
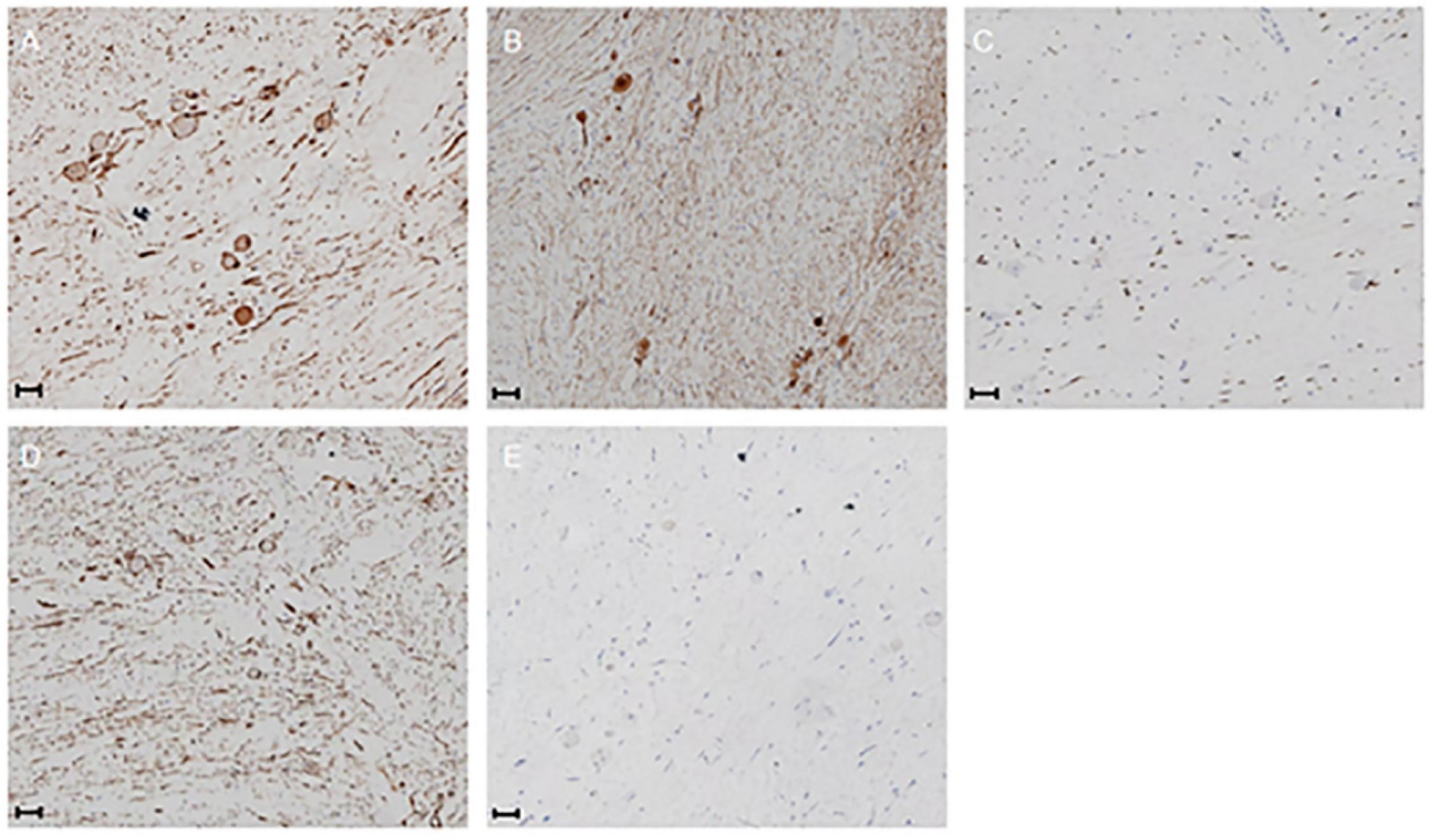
20×. **(A)** S-100(+) **(B)** Syn (+) **(C)** SOX10(+) **(D)** Vim (+) **(E)** Ki-67 < 5%. Scale bar  = 50 μm.

Microscopic examination revealed that the tumor cells were elongated and spindle-shaped, with some areas exhibiting myxoid changes. Additionally, mature ganglion cells were observed (Figure 5 demonstrates). Immunohistochemical analysis further corroborated the diagnosis of GN (Figure 6 demonstrates). We summarized the immunohistochemical results in Table 2.

**Table 2:** Immunohistochemistry.

Marker	Result
Vim	+
S-100	+
SOX-10	+
CD34	−
SMA	−
CK-P	−
Syn	+
Ki-67	+, <5%

## 3 Postoperative outcomes

On the first day after operation, the patient rested in bed and was treated with antibiotics to prevent infection (within 3 days after operation), proton pump inhibitors to inhibit acid and fluid replacement; On the other hand, because of the postoperative incision, pain relief is also necessary (especially on the day and the first day after surgery), the patient was given analgesics; On the second day, the catheter was removed and the patient got out of bed; Considering the patients' paroxysmal atrial fibrillation, heart failure and other basic diseases, we also reviewed the cardiac indicators on the second day after operation. The results showed that cTn I was 3.208 ng/mL and BNP was 210.79 pg/mL. Fortunately, the patient did not have any special clinical manifestations.

In terms of intestinal management, due to the proximity of the patient's tumor to the stomach (Figure 2B demonstrateds), a gastric tube was inserted three days prior to the operation to prevent interference with the surgical field of vision and to alleviate postoperative abdominal distension. The gastric tube was removed on the second day post-operation after adequate ventilation, and a liquid diet was initiated. On the third day, a semi-liquid diet was introduced. Ultimately, the patient recovered and was discharged on the fifth day following the operation.

After recovery and discharge, we conducted telephone follow-up at 1, 3, and 6 months postoperatively. The patient recovered well after the operation and did not experience any complications or require hospitalisation for further treatment.

## 4 Discussion

GN originates from embryonic undifferentiated cells of the neural crest and is classified as a benign differentiated tumor of the sympathetic nervous system (6). While it can occur in any region of the sympathetic nervous system, it is most frequently found in the abdomen, with 52% located in the paravertebral retroperitoneal sympathetic ganglion, 39% in the posterior mediastinum, and 9% in the pelvis or neck (7, 8). Within abdominal GN, 49% arises from the adrenal gland, while 51% originates from outside the adrenal gland (8). GN is a subtype of neuroblastic tumors (NTs), which can be categorized into four types based on morphological characteristics: neuroblastoma, ganglion neuroblastoma (mixed type), ganglion neuroblastoma (nodular type), and GN (9). Histologically, GN is characterized by a predominance of benign and well-differentiated cells compared to ganglion neuroblastoma and neuroblastoma. The tumor typically contains mature ganglion cells, spindle cells, and a collagen matrix, and usually presents as a well-defined tumor encapsulated by fibrous tissue (10). GN may be multifocal or associated with other independent neurogenic or neuroendocrine tumors. Although catecholamine synthesis is a consistent feature of nearly all neurogenic tumors, the clinical symptoms resulting from hormone excess in GN are exceedingly rare (9).

### 4.1 Anatomical features and diagnostic challenges

#### 4.1.1 Anatomical variations

GN often lacks distinct clinical symptoms and exhibits a low incidence, making diagnosis challenging. Most patients are identified incidentally through auxiliary examinations, while a minority present with clinical symptoms resulting from tumors compressing adjacent organs. These characteristics often lead to the discovery of tumors at a larger size. Kirchweger et al. (11) conducted a literature review from 1957 to 2020 and reported that 10 of the 13 cases of thoracic and abdominal GN had tumor sizes exceeding 10 cm, with the largest measuring 23 cm. This particular patient was treated for persistent left chest pain, and during surgery, it was revealed that the tumor had invaded the superior sulcus of the lung, adhered to the posterior and medial aspects, and encased the thoracic aorta (12). Regardless of whether GN occurs in the mediastinum or retroperitoneal space, the dense arrangement of thoracic and abdominal organs and blood vessels complicates matters; large tumors typically exhibit significant adhesion to surrounding structures. This can lead to misdiagnosis during the diagnostic process. Current cases typically present similar challenges, with the tumor located on the left side and clearly adhering to the blood vessels (Figure 1 demonstrates). Notably, the prevalence of left IVC is low. The incidence of left IVC in the general population is 0.2%–0.5% (13), but reaches 3.8% in patients with retroperitoneal tumors. However, we confirmed that the blood vessels involved in tumor adhesion were ectopic IVC through three-dimensional vascular reconstruction (Figure 2A demonstrates) prior to the operation, which provided the surgeon with adequate psychological preparation.

#### 4.1.2 Imaging differentials

GN can originate from any part of the sympathetic nervous system and often adheres to surrounding organs and blood vessels, resulting in a particularly complex anatomical structure. Fortunately, tumors originating from nerve tissue exhibit certain characteristic manifestations in imaging diagnosis. A review of imaging features from previous cases indicates that GN typically presents as homogeneous or slightly inhomogeneous low-density lesions on CT. Notably, GN may occasionally display local calcification, which can lead to misdiagnosis as a dermoid cyst. The histopathological features of GN elucidate its imaging findings: the mucus matrix appears as low density on CT and exhibits characteristic high signal intensity on T2-weighted MRI; nerve cell bodies and nerve fibers present as areas of increased density on CT, while they display low signal stripes on T2-weighted MRI. On T1-weighted MRI, GN may show edge enhancement due to the capsule; however, dense adhesions may obscure the capsule's characteristics, resulting in progressive enhancement on MRI during the delayed phase (14). When vascular invasion is present, GN must be differentiated from hypervascular tumors such as paraganglioma-derived tumors (15) and schwannoma-derived tumors (16, 17). Paragangliomas, such as pheochromocytomas, typically exhibit significant abnormalities on preoperative hormone tests and are often associated with uncontrolled hypertension; on imaging, the CT value of GN is generally less than 40 HU, while paragangliomas demonstrate marked enhancement on CT scans (15). In contrast, schwannomas exhibit heterogeneous enhancement and attenuation on imaging, whereas GN shows homogeneous enhancement and attenuation (16).

### 4.2 Optimization of surgical strategy

In many cases, complete tumor resection is the preferred treatment modality. As previously mentioned, most patients present with a significant tumor volume at the time of diagnosis, which is often closely associated with surrounding organs and blood vessels. In severe instances, this may lead to spinal cord compression (18). Consequently, surgical procedures can be particularly challenging and may necessitate multidisciplinary collaboration. The dissection of the tumor from the surrounding adhesions of organs and blood vessels requires meticulous technique.

For retroperitoneal tumors, traditional open surgery is associated with several disadvantages, including large incisions, intraoperative side injuries, prolonged recovery times, and potential complications. Consequently, clinicians face significant challenges in perioperative management. However, with the recent advancements in minimally invasive technology, laparoscopic treatment of retroperitoneal tumors has been successfully documented (19, 20). For instance, Chatelet et al. reported the resection of a 17 × 8 × 6 cm retroperitoneal tumor using a laparoscopic approach in 2018 (21). Additionally, Ahn et al. conducted a comparative study involving 20 patients with retroperitoneal tumors who underwent laparoscopic surgery and 14 patients who underwent open surgery. Their findings indicated that laparoscopic surgery resulted in less blood loss, shorter operation times, and reduced postoperative hospital stays. Remarkably, even in cases where the tumor volume is large or the tumor adheres to adjacent vascular structures, laparoscopic retroperitoneal tumor resection remains feasible (22). Early studies have demonstrated that transperitoneal laparoscopic surgery is a superior treatment option for GN, as it provides clearer anatomical landmarks and reduces the risk of major vascular injury (15). With the advancement and clinical application of surgical robotics, their benefits—such as three-dimensional visualization, enhanced degrees of freedom, elimination of the fulcrum effect, reduction of physiological tremor, and improved flexibility have been increasingly validated. Since the introduction of the Da Vinci surgical system into urology in 1999, robotic systems have been used in various urological procedures. Compared with conventional laparoscopy, the robotic approach reduces blood loss by 38% in vascularized retroperitoneal tumors (23). The utilization of robot-assisted operations is increasingly recognized for its precision, safety, and stability, surpassing traditional laparoscopic surgery. These advanced techniques can significantly minimize damage to surrounding tissues and decrease the incidence of complications (24, 25). However, from a clinician's perspective, it is essential not to rely solely on robotic technology. On one hand, the high medical costs and the extensive training required for physicians must be taken into account. On the other hand, as noted in Ahn et al.'s study, malignant lesions often require more extensive resections to ensure safe surgical margins (22). While we appreciate the benefits of minimally invasive surgery, we must not compromise patient welfare. Furthermore, there is a pressing need for more prospective controlled studies with larger sample sizes to validate these findings.

Large tumors located in the narrow regions of the retroperitoneal space can fully utilize the manipulator's degrees of freedom, enabling 360° rotational maneuvers without dead angles. Additionally, the surgical robot provides a clear three-dimensional visual effect, allowing surgeons to accurately differentiate tumors from surrounding organs, tissues, and blood vessels, thereby minimizing secondary injury. Furthermore, it is important to note that the robotic arm significantly reduces manual tremor in surgical instruments. This tremor-filtering capability greatly reduces the risk of injury, particularly during the dissection of major blood vessels such as the IVC. Recent meta-analysis results regarding robot-assisted surgery for renal tumors complicated by IVC thrombosis indicate that, in comparison to open surgery, robot-assisted surgery is associated with reduced blood loss and a lower requirement for blood transfusions, as well as a decreased complication rate and shorter hospital stays (26). Robot-assisted treatment of retroperitoneal tumors effectively separates the tumor from adjacent major blood vessels, allowing for better control of bleeding, even in close proximity to the IVC, abdominal aorta, porta hepatis, and porta renal (27). Furthermore, research on renal cell carcinoma complicated by IVC thrombus suggests that employing “minimal-touch” techniques can help free the IVC while minimizing vascular damage. In this context, the primary traction is applied to the tissues surrounding the IVC rather than the IVC itself. However, in robotic surgery, the utilization of a 30° up-down robotic lens and micro-wrist instruments facilitates precise control over blood vessels and surrounding tissues, thereby minimizing the manipulation of the IVC and adjacent structures (28). However, despite the robotic system's ability to substantially lower the risk of secondary injuries, in cases involving IVC vascular invasion, it is advisable to perform preoperative three-dimensional vascular reconstruction to assess the contact angle (>180° in this case) and to prepare vascular repair materials if necessary, in anticipation of potential intraoperative vascular injuries. In this case, the tumor exhibited no significant supply vessels. Consequently, we employed the robot's 3D high-definition vision to accurately identify and occlude the supply vessels when they were free. Subsequently, we ligated the small supply vessels associated with the tumor and meticulously freed the tumor before proceeding to its complete removal.

Retroperitoneal tumors, particularly those of adrenal origin, necessitate careful consideration of hormone secretion, which is a significant factor contributing to severe complications during the perioperative period. In this case, we conducted a thorough assessment of adrenal-secreted hormones for the patient prior to surgery. The results indicated a slight elevation in aldosterone levels in the supine position and during the early morning, with all other hormone levels remaining within normal ranges. Although these findings did not suggest any significant abnormalities, we opted to expand the patient's fluid volume preoperatively to enhance blood pressure stability and ensure intraoperative safety. Furthermore, we conducted a comprehensive postoperative review to mitigate the risk of adrenal insufficiency due to decreased hormone levels, which were found to be normal following surgery.

### 4.3 Progress of pathological diagnosis and prognosis

In the pathological diagnosis of GN, nerve fibers are observed to be arranged in a wavy manner under the microscope, with mature ganglion cells scattered throughout the tissue. Immunohistochemical staining, including markers such as S-100, Vim, neuron-specific enolase, neurofilament, and myelin basic protein, is typically positive, further confirming the neural origin of the tissue (14). Postoperative pathology is a critical focus for clinicians.

#### 4.3.1 Histopathological characteristics

Among the various types of NTs, GN is characterized by the highest degree of benignity and must be differentiated from ganglioneuroblastoma (mixed type, nodular type) and neuroblastoma. Notably, neuroblastoma is a fatal malignant tumor that accounts for 10% of pediatric cancers (29). The identification of these tumors primarily relies on the development of Schwann's matrix and the mitosis-karyorrhexis index (MKI). After pathological section staining, neuroblastoma under the microscope showed less Swan's matrix; ganglion neuroblastoma (mixed type) showed Doschwan's matrix; ganglion neuroblastoma (nodular type) is characterized by complex, that is, more schwann's matrix/schwann's matrix and less schwann's matrix coexist; GN is dominated by Schwann's matrix, and there are fully mature ganglion cells covered by satellite cells (30). Neuroblastoma is divided into three subtypes: undifferentiated, poorly differentiated, and differentiated, while GN is divided into two subtypes: maturing and mature (30).

In this case, mature ganglion cells were found by pathological examination. Immunohistochemical results showed that SOX10 expression was detected in 10,000 cells, Syn was located in neurosecretory granules, and Masson trichrome staining revealed a characteristic collagen matrix distribution, consistent with the immunophenotype of typical GN. Notably, while biopsy has a high accuracy rate (31), its diagnostic outcomes typically do not alter the treatment plan, rendering it often unnecessary in clinical practice. Following surgical resection, the prognosis for GN is generally favorable (6). For the majority of patients, R0/R1 resection is curative, with only a few cases of recurrence reported during the three-year follow-up period, which is relatively uncommon (32, 33). Furthermore, a limited number of cases of malignant transformation post-surgery have been documented, but these occurrences are exceedingly rare (34). Therefore, long-term follow-up remains essential.

## 5 Conclusion

The case of GN with ectopic IVC has not been previously reported and should be considered in the differential diagnosis of retroperitoneal space-occupying lesions. The diagnosis and treatment of retroperitoneal GN with vascular invasion necessitate attention to the following points: 1. Imaging characteristics: GN exhibits unique pathological features and demonstrates strong resolution in imaging. The specific manifestations are as follows: (a) plain CT shows relatively low density; (b) T2-weighted images reveal a “vortex sign”; (c) it tends to encase large blood vessels, although not exclusively; (d) enhanced CT and MRI indicate delayed progressive enhancement. Therefore, enhanced CT combined with MRI diffusion-weighted imaging can improve the accuracy of preoperative GN diagnosis and clearly delineate the degree of adhesion between the tumor and surrounding blood vessels. 2. Surgical methods: Robot-assisted laparoscopic approach offers significant advantages in managing vascular adhesions, including three-dimensional visualization, a high degree of maneuverability, elimination of the fulcrum effect, suppression of physiological tremor, and enhanced flexibility, thereby reducing the risk of vascular injury. 3. Preoperative evaluation: For ectopic IVC, preoperative CTA/MRA three-dimensional reconstruction techniques are essential to clarify the source, course, and anatomical relationship of blood vessels adhered to the tumor. In summary, high-quality imaging evaluation and meticulous surgical planning are crucial for successful treatment. This case confirms that R0 resection can be achieved through a precise robot-assisted laparoscopic approach, even in the presence of vascular anatomical variations. This case highlights the importance of recognizing vascular anomalies in preoperative planning for retroperitoneal masses. Moreover, very few patients experience recurrence after surgery; therefore, it is recommended to have annual contrast-enhanced MRI for 5 years postoperatively.

## Data Availability

The raw data supporting the conclusions of this article will be made available by the authors, without undue reservation.
